# P-1882. Curriculum-Based Online Medical Education Effectively Improves Clinician Knowledge, Competence, Confidence, and Performance with Integrating Antimicrobial Stewardship When Diagnosing and Managing Suspected Pneumonia with Available Molecular Rapid Diagnostic Tests

**DOI:** 10.1093/ofid/ofaf695.2051

**Published:** 2026-01-11

**Authors:** Arun S Nair, Maria B Uravich, James Martorano

**Affiliations:** Medscape Education, TARRYTOWN, New York; Medscape Education, TARRYTOWN, New York; Medscape EDucation, New York, NY

## Abstract

**Background:**

This study assessed the impact of online CME on knowledge, competence, confidence, and performance related to molecular rapid diagnostic tests (mRDTs) & antimicrobial stewardship (AMS) in diagnosing and managing suspected pneumonias.

**Methods:**

The CME curriculum included two online activities. The 1st activity featured 5 video-based micro-CME chapters by multidisciplinary experts, with learners selecting chapters. Education effects were measured using a repeated pairs pre-/post-assessment design, with participants as their own controls. McNemar’s tests (*P* < .05) assessed mastery (correct decisions or improved confidence), and a confidence-based assessment (CBA) evaluated learning changes. The 2nd activity was a 30-minute video discussion with slides. Real-world performance was assessed via a survey 30–60 days post-education, capturing practice changes & barriers. Data across activities were collected from 8/2024 - 3/2025.

**Results:**

As of March 2025, the curriculum reached ∼50,000 learners, primarily targeting Infectious Disease (ID) Specialists, Pulmonologists, Critical Care (CC) Specialists. Data from those who completed pre-/post-assessments for the first activity showed significant learning improvements (*P* < .001). Overall, 65% of ID Specialists (n=402), 61% of Pulmonologists (n=445), and 63% of CC Specialists (n=230) improved knowledge, competence, and/or confidence related to incorporating and interpreting mRDTs when diagnosing & managing suspected pneumonia.

For the 2nd activity, 35 learners completed the survey showed 96% made or reinforced practice changes due to education. Top changes included:

• Interpreting mRDT results for pneumonia (69%)

• Using mRDTs to confirm diagnosis (54%)

• Using semiquantitative results from mRDTs & collaborating across specialties to optimize AMS (51%)

Barriers to practice included:

• Lacking familiarity with mRDTs

• Difficult to obtain bronchoalveolar lavage specimens

• Insufficient training for colleagues in other professions

• Lack of consensus guidelines for mRDT use

**Conclusion:**

These findings demonstrate that online CME/CE promotes adoption of AMS-related practices for diagnosing & managing suspected pneumonia with mRDTs. Future education should address identified barriers to further enhance practice changes.

**Disclosures:**

All Authors: No reported disclosures

